# Evacuation behaviors and emergency communications: An analysis of real-world incident videos

**DOI:** 10.1016/j.ssci.2020.105121

**Published:** 2021-04

**Authors:** C. Natalie van der Wal, Mark A. Robinson, Wändi Bruine de Bruin, Steven Gwynne

**Affiliations:** a Department of Multi-Actor Systems, Delft University of Technology, the Netherlands; b Socio-Technical Centre, Leeds University Business School, Leeds, United Kingdom; c Sol Price School of Public Policy, Dornsife Department of Psychology, Schaeffer Center for Health Policy and Economics, and Center for Economic and Social Research, University of Southern California, United States; d Mövement Strategies, London, United Kingdom; e Lund Unviersity, Sweden

**Keywords:** Emergencies, Evacuation, Risk communication, Crowd behavior, Crowd management, Video analysis

## Abstract

Emergencies such as fires and terrorist attacks pose risks of injuries and fatalities, which can be exacerbated by delayed, ill-informed, or unmanaged responses. Effective emergency communication strategies could be used to better inform people and reduce these risks. This research analyzes videos of real-world emergencies to: (a) identify people’s observed behaviors that increase risk during evacuations, and (b) examine which emergency communication strategies might reduce risk behaviors. We analyzed 126 publicly available videos of emergency evacuations in different emergencies (e.g., fire, terror attack, evacuation alarm, perceived threat). We found evidence of three types of risk behaviors (delayed response, filming, running) and four emergency communication strategies (evacuation alarm, staff guiding people to exits, general prerecorded message, live announcement). Our analyses suggest that having staff guide people to exits is the most effective strategy for promoting faster and more effective responses. However, neither live announcements nor pre-recorded messages were associated with delayed responses, while evacuation alarms were associated with more delayed responses than other communication strategies. Although people filming the incident was unrelated to staff interactions, it occurred more with alarms sounding and prerecorded messages, suggesting that these emergency communications might not prevent filming. Compared to no communications, all emergency communication strategies reduced running during evacuations. We discuss the implications of this research for identifying effective emergency communication strategies and reducing risk-increasing evacuation behaviors.

## Highlights

• Videos were analyzed to examine which emergency communication strategies might reduce risk behaviors.
• We believe this is the first video analysis relating emergency communications to evacuation behaviors.
• Our analyses suggest that having staff guide people to exits is the most effective evacuation strategy.
• Evacuation alarms were associated with more delayed responses than other communication strategies.
• People filming the incident occurred more with alarms sounding and prerecorded messages might not prevent people filming the incident.
• Compared to no communications, all emergency communication strategies were associated with running during evacuations.
• Our main practical recommendation is to supplement traditional emergency alarms with guidance from staff.

## 1 Introduction

The outcome of an emergency incident is influenced by the nature and timing of people’s responses, which may include delaying evacuation, taking the familiar exit, running, and filming, all of which can increase risk and impede safe evacuation (Kobes et al., 2010a, Lovreglio et al., 2016, Purser and Bensilum, 2001). Interviews held with crowd safety experts from different fields suggest that, among others, the following three behaviors were experienced the most: delayed responses, running, and filming (Van der Wal, 2019). While delayed response to alarms or incidents is one of the most researched risk behaviors, filming and running can also be dangerous (Kobes et al., 2010b, Proulx and Sime, 1991, Shiwakoti et al., 2017, Shiwakoti et al., 2020). A ‘risk behavior’ is defined as a behavior that exposes someone to risk, which may result from people’s risk perceptions (Kinateder et al., 2015).

Evacuation research has focused mainly on risk behaviors during evacuations from fires and terrorism (Fahy and Proulx, 2005, Kobes et al., 2010a, Kobes et al., 2010b, Lovreglio et al., 2019, McConnell et al., 2010), with the former causing more fatalities (Ritchie, 2018). Videos of fire and terrorism emergencies and eye witness reports have suggested three risk behaviors that occur during emergency evacuations (Donald and Canter, 1992, Galea et al., 2012, Grosshandler et al., 2005, Kobes et al., 2010a, McConnell et al., 2010, Proulx and Fahy, 1997). First, people may be slow to evacuate (McConnell et al., 2010, Proulx and Fahy, 1997). For example, people may take up to 9 min longer to respond to evacuation alarms in residential drills than in office drills, which may be explained by alarm audibility, occupant training, and the presence of fire wardens (Proulx & Fahy, 1997). Second, people have been observed running during evacuations, which may increase the number of collisions (Burroughs and Galea, 2015, Galea et al., 2012, Grimm et al., 2014, Grosshandler et al., 2005, Kobes et al., 2010b, Makinoshima et al., 2020, McConnell et al., 2010). Third, filming with smartphones or other cameras during incidents, such as fires and shootings, has also been observed (Antony and Thomas, 2010, Aucoin, 2019). Given the prevalence of mobile technology and social media use, it is likely that this will only increase.

Risk behaviors typically arise during emergency evacuations due to a lack of situational awareness or a lack of guidance – leading to a misunderstanding of the severity of the situation (Kobes et al., 2010a, Kinateder et al., 2015). People might not realize that there is an emergency when they do not receive or understand evacuation instructions or fail to perceive the threat cues (Nilsson and Johansson, 2009, Proulx and Fahy, 1997, Shiwakoti et al., 2018). Socio-cultural differences and variations, such as nationality, age, mental abilities, have also been observed to influence emergency responses; although most people generally respond in a recommended manner (Galea et al., 2011, Grimm et al., 2014, Kholshevnikov et al., 2009, Shields et al., 1999).

### 1.1 Risk behaviors and their relation to emergency communication.

#### 1.1.1 Delayed response

Emergency communications aim to reduce risk by enhancing situational awareness of the incident and of viable responses, but may vary in their effectiveness (Proulx, 1999, Lovreglio et al., 2016). In a seminal piece of work, evacuation time at an underground train station during an unannounced drill took up to 9 min with an alarm only, but was reduced to between 1 and 7 min with the addition of a recorded or live voice alarm, staff directions, or visual display information (Proulx & Sime, 1991). Other studies have shown that pre-recorded or live voice alarms produce a quicker response than alarm sounds only (Purser, 2010), and that the presence of staff members can have a significant effect on improving response time and exit choice (Samochine et al., 2005).

The first priority of emergency communication is to alert people to evacuate, when evacuation is appropriate (Proulx, 1999). In some cases, evacuation instructions will also be accompanied by explanations of why the evacuation is needed, through a voice alarm or staff present at the scene (Gwynne et al., 2009, Proulx and Sime, 1991, Purser, 2010).

Most existing evidence of evacuee decision making comes from unannounced drills, and surveys and interviews with eye witnesses of real incidents (Grimm et al., 2014, Huang et al., 2015, Kobes et al., 2010a, Kuligowski et al., 2013, Lovreglio et al., 2016, Omori et al., 2017, Proulx et al., 1995, Schmidt and Galea, 2013). Early research on human behavior in response to fires (Tong & Canter, 1985) adopted a now outdated ‘physical’ and ‘panic’ approach, in which people were viewed as ‘ball bearings’ dominated by physical factors and incapable of rationally processing information. It is now understood that social and physical factors interact, with social interactions influencing evacuation time from buildings (Nilsson and Johansson, 2009, van der Wal et al., 2017).

Delayed responses to an incident can reduce the time available to reach safety and reduce options available to those evacuating, due to, for example, fire and smoke blocking previously available routes (Aguirre et al., 2011, Fahy and Proulx, 2005, Grosshandler et al., 2005, McConnell et al., 2010). Delayed responses to the Station Nightclub fire in Rhode Island in 2003 led to 100 fatalities, exacerbated by crowding at the main exit (Aguirre et al., 2011, Grosshandler et al., 2005). Delayed response times of up to 11 min in WTC Tower 1 and up to 25 min in WTC Tower 2 were reported in the 9/11 terrorist attacks in New York in 2001, mainly due to people not recognizing the risk (Fahy and Proulx, 2005, McConnell et al., 2010). Delayed responses also occur when shows or sports events continue despite an emergency, or when a fire alarm is misinterpreted as a prank or drill (Proulx, 1999). To maximize the likelihood of a quick response, people have to be made aware, clearly and unambiguously, that action is required (Proulx, 1999).

Databases of response times from multiple evacuation experiments or real incidents indicate delayed responses can vary as a result of the notification people receive, with alarms used alone generally eliciting slowest responses compared to warnings by staff, voice announcements, and visible smoke (Fahy and Proulx, 2001, Lovreglio et al., 2019). Our secondary analysis of Lovreglio et al.'s (2019) review found that, across all drills and fire incidents, response times were faster for a prerecorded voice message (0.68 min) and voice alarm (0.87 min) than for an alarm sound only (1.68 min), though each of these strategies led to faster responses than no alarm at all (27.97 min).

Providing information to occupants as early as possible during emergencies will inform their understanding of the situation and enable them to respond faster (Proulx, 2001). However, Benthorn and Frantzich (1999) found that people do not always realize that alarms and pre-recorded messages require evacuation. In their field study, participants in a retail store heard a fire alarm followed by a verbal evacuation announcement. The majority interpreted the verbal announcement correctly as suggesting a serious problem, a need for evacuation, or fire. However, they also mistook the alarm bell for an ordinary unspecified warning or problem, with only a minority realizing that there was a fire or another situation requiring evacuation (Benthorn & Frantzich, 1999).

Voice messages and the presence of trained staff who assist those evacuating have also been found to improve evacuation time (Samochine et al., 2005, Shields and Boyce, 2000). In an unannounced retail store evacuation, customers relied on the staff to quickly guide them to the exits (Shields & Boyce, 2000). The presence of trained staff has also been effective in multiple residential and office building evacuation drills, and helped evacuees to see the need to respond to fire alarms (Proulx & Fahy, 1997). Delays tended to be caused by, among other things, poor alarm audibility, misinterpretation of the situation, not seeing others evacuate, and absence of fire wardens. Furthermore, the occupants of the office buildings evacuated faster than those of the residential buildings, because they had received fire drill training (Proulx & Fahy, 1997). However, because participants were aware that an evacuation drill would be performed (at an unspecified time and date), results showed that only 20–25% of participants believed it was a real fire emergency (Proulx & Fahy, 1997).

#### 1.1.2 Running

Running can lead to falls and increased obstructions and collisions, resulting in injuries (Harding et al., 2010, van der Wal et al., 2017). Running during evacuations has been observed in different emergencies and field studies, including hotel evacuations, subway stations, primary schools, or other buildings (Hamilton et al., 2017, Kobes et al., 2010b, Sørensen and Dederichs, 2015, Yoon et al., 2013). Running has also been observed in terrorist attacks or active shooter scenarios (Anderson et al., 2007, Iqbal, 2015). People may start running when they observe staff or security personnel running (Proulx, 1999, Sandberg, 1997). Rapid evacuation can produce injuries in addition to those produced by the original incident itself (van der Wal, 2019). However, we could not find literature on the effects of emergency communication on running.

#### 1.1.3 Filming

People might record emergency conditions in an attempt to document important events (e.g. as a 'public eye') or to simply store material for their records (e.g. as a 'disaster tourist') ‘public eye’ to irresponsible ‘disaster tourist’ (Allan & Peters, 2015). Digital journalism or ‘smartphone bystanders’ is a relatively new phenomenon enabled by ubiquitous mobile technology (Allan and Peters, 2015, Andersson and Sundin, 2016). We found no specific existing literature on people filming during evacuations. However, videos of emergency evacuations are widely available, providing a useful data source for investigating risk behaviors during videos of actual emergency incidents (Van der Wal, 2020).

### 1.2 Current study

In this study, we focus on publically-available videos of ongoing emergency evacuations—situations in which people are or should be evacuating immediately—involving the following incidents: fire, terrorist attack, shooting, tornado, hurricane, storm, crowd congestion at an event, sinking ship, crowd scare, and general alarm sounding. We examined videos that were collected online, from YouTube and news sites, including smartphone videos from the general public, CCTV footage, or live TV. The benefit of using videos is that there is no reliance on potentially distorted memories or self-reports. These methodological advances increase the accuracy and realism with which emergency behaviors can be examined.

We examine three risk behaviors (*delayed response, filming, running*), four emergency communications (*alarm sounding, general prerecorded message, staff guiding people to exits, live message),* and one contextual variable *(perceivable threat)* in these videos of real-world incidents, as these were observable and discussed in the research literature. Our specific research questions are as follows:

1. How frequent are risk behaviors in the evacuation incidents captured in our video samples (i.e., delayed response, filming, running)?
2. How frequent are emergency communications in our video samples (i.e., evacuation alarm, general prerecorded message, staff guiding people to exits, or live announcement)?
3. How do these emergency communications relate to these risk behaviors?

## 2 Method

### 2.1 Data collection

We collected 126 publicly available videos, by searching for emergency evacuations in the Disasters Database (Still, 2020) and on YouTube. Our inclusion criteria focused on selecting videos in which: (1) people were visibly evacuating, or (2) people should be evacuating, as there was an indication of an emergency, such as the alarm sounding or a visible threat such as a fire. We excluded videos where no evacuating people were visible, including aftermath images or news items, ‘mosh pits’ at music gigs, crowd collapses, simulation videos, and crowd surges into shops or malls, instructional videos on how to evacuate, videos showing drills, staged evacuations, and news videos with no evacuation footage from the actual event. Overall, we included 80 videos from the Disasters Database based on these inclusion and exclusion criteria.

We searched YouTube between 1–10 March 2018 and 1–10 October 2018, using the search terms ‘evacuation’, ‘fire evacuation’, ‘crowd disaster’, ‘terror attack’, and ‘immediate evacuation’. We viewed the first 50 videos returned for each search term. Additionally, we used a snowball sampling approach where we also reviewed the first 20 videos that YouTube indicated as related recommendations. We continued this process for 120 h until no new videos were found, resulting in a set of 85 videos from YouTube.

This search resulted in 165 videos, comprising 80 from the Disasters Database and 85 from YouTube. Next, we screened these videos to remove any duplicates, including videos of the same incident—which were only included if the people and location in the recording were different. The final sample included 126 videos, including 54 from the Disasters Database and 72 from YouTube, representing 107 different incidents. The videos were filmed by the following sources: 83% by visitors, evacuees, bystanders, or vloggers, 12% by journalists for TV programs, 5% by CCTV or webcam. The videos and their descriptions are stored in an online repository (van der Wal, 2020).

### 2.2 Coding

Two independent coders evaluated each video for the presence or absence of the following three risk behaviors: (1) *Delayed response,* (2) *Focused on filming instead of evacuating,* (3) *Running;* and then for the presence or absence of the following four emergency communications: (1) *Evacuation alarm sounding,* (2) *General prerecorded message,* (3) *Staff guiding people to exits,* (4) *Live announcement*; and one contextual variable: *perceivable threat*.

After training on a randomly selected 20% of the videos, another randomly selected subset of 20% of the videos was coded independently by both coders. Training was performed via observing the videos together and explaining how to code each behavior. The two independent coders agreed on 91% of the codes, reflecting strong inter-rater reliability (*kappa* = 0.81), then resolved disagreements through discussion (McHugh, 2012). The remaining videos were evaluated by one coder.

We tested the relationship between the frequency of each of the four emergency communications (our predictor variables) and each of the three behavioral responses (our outcome variables) with Chi-square statistical tests. In addition, we conducted logistic regressions examining all four emergency communications as predictors of each of the three (binary) behavioral response outcomes.

The coding scheme used the following operational definitions for the three behavioral categories and the four communication categories below.

#### 2.2.1 Behavioral categories

**Fig. 1:**
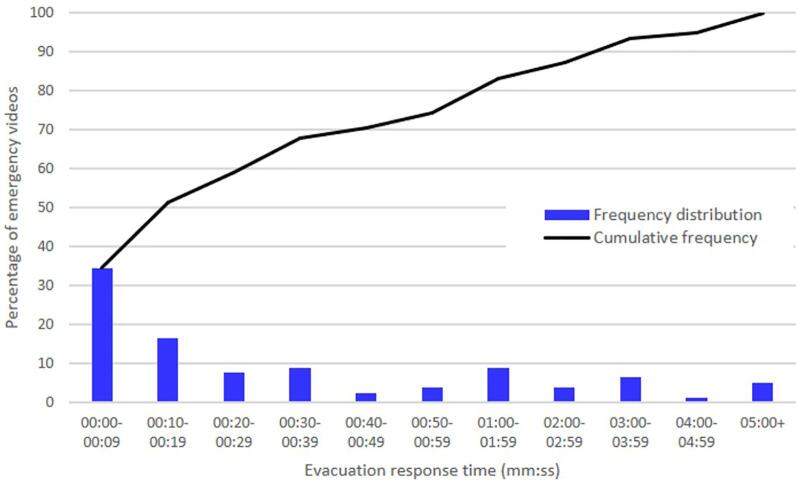
Distributions of response times to incidents for the majority of people in the emergency videos before commencing evacuation.

*Delayed response.* Following previous research, the operationalization of delayed response relied on both quantitative assessments of response times and qualitative assessments of delaying behaviors (Gwynne and Boyce, 2016), as described below. The quantitative assessment was based on the number of seconds that elapsed between the first indication of the incident and the point at which more than half of the people visible in the video had commenced their evacuation. The full distribution of response times across the emergency videos is provided in Fig. 1. The quantitative assessment of delayed response was dichotomous, and reflected whether the response time was 30 s or longer (vs. not). (see Appendix for analyses). Previous evacuation research has indicated that 30 s is a key threshold, as it is close to the modal response time in many studies and is sufficiently quick to enable safe evacuations generally (Purser & Bensilum, 2001). For evacuation time to be measured accurately, the incident had to start during the video, a criterion met by 78 of the 126 videos. However, even when the incident was already unfolding at the beginning of the video, the response was still categorized as ‘delayed’ if it took 30 s for the evacuation to be observed in the video.

We also incorporated qualitative observations of delayed response. These qualitative observations included congestion slowing movement towards the exit, someone picking up belongings or waiting for another adult before evacuating, or other observable reasons for not taking the immediate route to the emergency exit (e.g., asking for information or going to find more information without evacuating) (Gwynne and Boyce, 2016).

The gamma correlation between the quantitative and the qualitative measure of delayed response was 0.76, *p* <0.001, suggesting agreement.

*Filming* was coded as present (vs. absent) if at least one person was focused on filming during the incident. Specifically, we defined “focused on filming” as: (1) filming without moving in the evacuation direction (e.g., turning the camera round), (2) filming while standing still, or (3) talking about what is happening and making efforts to film these features while walking.

*Running* was coded as present (vs. absent) if at least one person was running during the incident. We defined “running” as sustained movement beyond walking speed where both feet leave the ground simultaneously during each stride.

#### 2.2.2 Communication categories and contextual variable

*Evacuation alarm* was coded as present (vs. absent) when an alarm signal was sounding.

*Staff guidance* was coded as present (vs. absent) if staff members were giving people verbal or gestural instructions about what to do. *General prerecorded message* was coded as present (vs. absent) when a general prerecorded message could be heard. *Live announcement* was coded as present (vs. absent) when a live message could be heard in which a member of staff was giving live updates or giving evacuation instructions. Live announcements could be distinguished from general prerecordings because they tended not to repeat or have pauses. *Perceivable threat* was coded as present (vs. absent) when there was an observable cause for alarm, including an observable fire, shooter, screaming people, or information from staff about the incident*.*

## 3 Results

### 3.1 Research questions 1 and 2: How frequent are emergency communications and risk behaviors in evacuations?

Table 1 shows the frequency of the three risk behaviors, and of the four emergency communications, as observed in our videos. Filming occurs most often (56%), followed by running (48%), then delayed response (35%). Each emergency communication occurs in less than a third of incidents: evacuation alarm (33%), staff guidance (32%), general pre-recorded message (16%), live announcement (11%).

**Table 1:** Frequency of risk behaviors and emergency communication strategies observed in the videos.

Action	Number (%) of videos (out of 126)	Description
**Risk behaviors**		
Filming	70 (56%)	A person is focused on filming instead of evacuating.
Running	60 (48%)	A person is running.
Delayed response –qualitative and quantitative*	44 (35%)	The majority (>50%) of visible people delay moving in the most efficient way towards the exit (due to congestion, collecting belongings, waiting for another adult, or a slow response > 30 s).
**Emergency communication**		
Evacuation alarm	41 (33%)	An alarm (a bell or tone) can be heard.
Staff guidance	40 (32%)	Staff are giving people instructions about what to do.
General prerecorded message	20 (16%)	A general prerecorded message can be heard.
Live announcement	14 (11%)	A live message can be heard in which a member of staff is giving live information about what is happening and/or instructions about what to do.

* Delayed response was analyzed both quantitatively and qualitatively (Gwynne and Boyce, 2016, Purser and Bensilum, 2001), as described in the Method section

### 3.2 Research question 3: How do emergency communications relate to delayed response, filming, and running?

#### 3.2.1 Delayed response

The Chi-square tests show that this risk behavior was more likely when the evacuation alarm was present and less likely when staff were guiding people to exits, but not more or less likely when there was a prerecorded or live announcement (Table 2). This risk behavior was less likely when a perceivable threat was present (Table 2). The logistic regression, which examined the independent relationships of communication strategies with delayed responses, showed that staff guidance significantly decreased the odds of delayed response behavior by 0.33 (Table 3). The other communication strategies (evacuation alarm, general prerecorded message, live announcement), and the situational context (a perceivable threat) did not have independent relationships with delayed response, however (Table 3).

**Table 2:** Frequency of risk behaviors when emergency communications are not present vs. present.

Emergency communication or contextual variable (perceivable threat)	Risk behavior when emergency communication not present	Risk behavior when emergency communication is present	Odds Ratio	Cramer’s *V*
***Delayed response***				
Evacuation alarm*	28.2%	48.8%	2.42*	0.20
General prerecorded message	34.9%	35.0%	1.00	
Staff guidance*	41.9%	20.0%	0.35*	0.21
Live announcement	35.7%	28.6%	0.72	
Perceivable threat*	47.2%	26.0%	0.39*	0.22
***Filming***				
Evacuation alarm***	44.7%	78.0%	4.40***	0.31
General prerecorded message*	50.9%	80.0%	3.85*	0.21
Staff guidance	58.1%	50.0%	0.72	
Live announcement	55.4%	57.1%	1.08	
Perceivable threat*	67.9%	46.6%	0.41*	0.21
***Running***				
Evacuation alarm***	64.7%	12.2%	0.08***	0.49
General prerecorded message***	54.7%	10.0%	0.09***	0.33
Staff guidance*	54.7%	32.5%	0.40*	0.21
Live announcement*	50.9%	21.4%	0.26*	0.19
Perceivable threat***	15%	71.0%	13.93***	0.56

*Note:* *** *p* < .001; ** *p* < .01; * *p* < .05.

**Table 3:** Logistic regression models predicting risk behaviors during evacuations.

	Model 1: *Delayed Response - Qualitative*	Model 2: *Filming*	Model 3: *Running*
***Evacuation alarm***			
Odds ratio	1.89	3.43*	0.17*
(95% CI)	(0.64–5.67)	(1.28–9.21)	(0.04-0.65)
*p*	0.25	0.04	0.01
***General prerecorded message***			
Odds ratio	0.43	1.75	0.73
(95% CI)	(0.12–1.57)	(0.43–7.07)	(0.11–4.71)
*p*	0.20	0.43	0.74
***Staff guidance***			
Odds ratio	0.33*	0.65	0.37
(95% CI)	(0.12-0.90)	(0.27–1.57)	(0.13–1.03)
*p*	0.03	0.34	0.06
***Live announcement***			
Odds ratio	1.01	1.39	0.43
(95% CI)	(0.24–4.24)	(0.39–4.97)	(0.09–2.11)
*p*	0.99	0.61	0.30
***Perceivable threat***			
Odds ratio	0.39	0.78	6.3**
(95% CI)	(0.15–1.02)	(0.31–1.93)	(2.22–17.87)
*p*	0.06	0.59	<0.01
			
Cox & Snell *R^2^*	0.117	0.109	0.310
Nagelkerke *R^2^*	0.16	0.15	0.41

*Note.* *** *p* < .001; ** *p* < .01; * *p* < .05. The odds ratios are presented with their 95% confidence intervals, meaning there is a 0.95 probability that the odds ratio will lie within this interval.

#### 3.2.2 Filming

The Chi-square tests suggest that filming is significantly increased by the evacuation alarm sounding or general prerecorded message, but not by the live announcement or staff guidance (Table 2). When examining the independent relationships of these communication strategies with filming and taking the situational context into account, we found that filming was less likely when a perceivable threat was present (Table 2). The results of the logistic regression suggest that filming was 3.43 times more likely when the alarm sounded, but was not additionally associated with the live announcement, general prerecorded message, staff guidance, nor perceivable threat (Table 3).^1^

#### 3.2.3 Running

The Chi-square tests suggest that running was less likely with the alarm sounding, general prerecorded message, live announcement, or staff guidance (Table 2). When examining the independent relationships of these communication strategies with filming and taking the situational context into account, we found that running was substantially more likely when there was a perceivable threat (Table 2). The results of the logistic regression indicated that running was much less likely (0.17) in the presence of an evacuation alarm sounding, but more likely when there is a perceivable threat (6.30) (Table 3).

## 4 Discussion

Emergency communication strategies aim to reduce the prevalence of risk behaviors during evacuations. Here, we analyzed 126 videos of evacuations from real-world emergency incidents to examine which communication strategies reduce risk behaviors. We found evidence of three types of evacuation behaviors (delayed response, filming, running) and four emergency communication strategies (evacuation alarm, staff guiding people to exits, general prerecorded message, live announcement). Our findings suggest that the most effective emergency communication strategy—for reducing risk behaviors such as a delayed response, filming, and running—was having staff guide people to exits, while the least effective communication is the sounding of an alarm by itself. Additionally, evacuation alarms and prerecorded messages increased the likelihood of filming behavior but staff guidance and live announcements did not. The presence of an evacuation alarm, live announcement, general prerecorded message, or staff guidance were all associated with less running during evacuations. Communication strategies were often implemented together. When considering the independent contribution of communication strategies and taking the situational context into account, the presence of a perceivable threat was associated with more running, but filming and delayed responses less likely.

To prevent delayed responses, staff guiding people to exits is the most effective communication. The sounding of an evacuation alarm by itself is the most ineffective communication, increasing the likelihood of delayed responses. These results correspond with our secondary analysis of Lovreglio et al.'s (2019) study, showing that for all drills and fire incidents the average response times for a voice alarm or prerecorded voice message were faster than for an alarm only or no alarm at all. The results also align with other findings that an alarm sounding is not always recognized to be indicating an incident (Proulx and Sime, 1991, Proulx, 2001) and people respond faster when there are staff guiding them to exits (Shields & Boyce, 2000).

None of the four emergency communications were observed to be effective at reducing filming behavior. Specifically, staff guidance and live announcements did not have a significant relationship with filming. Evacuation alarms and prerecorded messages actually were associated with significantly more filming behavior than no communication. However, taking the situational context and other communication strategies into account, the presence of a perceivable threat did decrease the likelihood of filming behavior. These results resonate with reported experiences from safety practitioners who indicated that filming is difficult to prevent and might be out of curiosity (Van der Wal, 2019). Possibly, filming is more likely when cues are unclear, as may be the case when evacuation alarms sound or general messages are given without specific information. To prevent running while evacuating, all four communications seem to be effective, however, suggesting that crowd professionals should choose whichever approach best integrates with their overall emergency communication strategy.

## 5 Strengths and limitations

We believe that a key strength of our research is the use of video recordings of actual behavior in real emergency incidents. Previous research has relied on field experiments, often by systematically varying communication strategies and contextual cues during emergency drills. Although emergency drills may have reasonable ecological validity compared to laboratory experiments, people’s behavior may still differ in response time or other risky behaviors from real-world emergency evacuations where there is danger and threat to life. (Robinson, 2016) Other previous research relies on self-reports. However, while self-reports can identify invisible cognitive processes, they are prone to recall accuracy which may bias findings. For instance, recall of situations can be biased by emotions and post-event information (Kaplan et al., 2016, Schacter and Loftus, 2013). More objective and accurate analysis of actual emergency behavior unfolding in real-time in videos of incidents can therefore substantially enhance our understanding. We believe that our video analysis complements the method of analyzing self-reports from survivors and evacuees, as part of an effective mixed-method strategy.

Another strength of our research is that it provides empirical evidence for a link between emergency communication and evacuation behaviors. First, we have identified which communications are most effective at facilitating faster responses and safer evacuations, and in which emergency circumstances, as we have discussed above with accompanying recommendations. Second, we hope that this research also stimulates further research into this important area by other researchers, by providing both findings on which to build and also a methodological protocol for studying such communication and behaviors in actual emergency incidents.

Our research has three main limitations. First, we could only analyze events for which videos existed and were identified in our search, so the sample of videos was not necessarily representative of the full range of real-world emergencies. Material may not have been uploaded to the public domain where it contained sensitive footage and where people have experienced the pressure to not film. Second, the videos may not have captured all people who experienced the event, or the full timeline of the event, with relevant behaviors potentially occurring off-camera. Third, as this was correlational research, we were unable to experiment systematically with the absence or presence of a communication strategy to examine its causal effects.

### 5.1 Implications and future research

The main practical recommendation arising from our findings is to supplement traditional emergency alarms with guidance from staff, either as additional verbal announcements or better still with guidance in person, to improve response times. While pre-recorded messages can help here, they run the risk of being misperceived as a false alarm or drill in the same way that regular alarms do. For these reasons, communication from humans that is clearly tailored to that specific situation and occurring in real-time is much more likely to facilitate faster evacuation.

A further practical recommendation is to improve public awareness of the danger of such risk behaviors in emergencies and encourage faster response times. A public awareness campaign could encourage people not to film and to evacuate quickly in emergencies, for instance.

Finally, our findings also have theoretical and methodological implications for future research. We have demonstrated that videos of real-world incidents can be systematically analyzed to examine the effectiveness of communication strategies. Future research could use video evidence to further assess the prevalence of delayed response, running, and filming in response to different communication strategies. It could also be used to examine correlations between response time and delaying behaviors, or between filming and congestion. Cultural factors can also play a role, for example in response times, which can be studied in future work (Galea et al., 2011). Our main research question here asked whether the risk behaviors occured, so future research can look at how many times they occur.

## 6 Conclusions

Our findings suggest that authorities should focus on staff intervention during incidents to reduce delayed evacuation responses and that any type of emergency communication shows promise for reducing running. Furthermore, we believe that more videos should be made available to researchers and safety practitioners to develop this promising methodological approach further. From these videos, we can learn which emergency communications occur, how frequently, and in which circumstances, and examine the responses to these factors. Such an approach will enable us to find solutions to prevent risk behaviors in evacuation and choose effective emergency communication strategies, which will ultimately prevent injuries and save lives.

## Figures and Tables

**Table 4:** Frequency of risk behaviors.

Risk behavior	Number of videos(out of 126)	Description
Delayed response –qualitative and quantitative*	44	The majority (>50%) of visible people delay moving in the most efficient way towards the exit (due to congestion, collecting belongings, waiting for another person, or a slow response >30 seconds).
Delayed response – quantitative*	32 (out of subset of 78 videos with observable response times)	The majority (>50%) of visible people have a slow response (≥30 seconds) to the incident before starting evacuation.

*Delayed response was analyzed both quantitatively and qualitatively, as described in the Methods section (Gwynne & Boyce, 2016; Purser & Bensilum, 2001)

**Table 5:** Relationships, odds ratios, and effect sizes between emergency communications and risk behaviors.

Emergency communication or contextual variable (perceivable threat)	Risk behavior when emergency communication not present	Risk behavior when emergency communication is present	Odds Ratio	Cramer’s V
*Delayed response (qualitative and quantitative)*				
Evacuation alarm	28.2%	48.8%	2.42*	.20
General prerecorded message	34.9%	35.0%	1.00	
Staff guidance	41.9%	20.0%	.35*	.21
Live announcement	35.7%	28.6%	.72	
Perceivable threat	47.2%	26.0%	.39*	.22
*Delayed response (quantitative)*				
Evacuation alarm	31.4%	59.3%	3.18*	.27
General prerecorded message	41.2%	40.0%	.95	
Staff guidance	45%	27.8%	.47	
Live announcement	40.8.%	42.9%	1.09	
Perceivable threat	56.7%	31.3%	.35*	.25

*Note:* *** *p* < .001; ** *p* < .01; * *p* < .05.

**Table 6:** Logistic regression models predicting risk behaviors during evacuations.

	Model 1: *Delayed Response – qualitative and quantitative*	Model 2: *Delayed Response - quantitative*
*Evacuation alarm*		
Odds ratio	1.89	2.50
(95% CI)	(.64-5.67)	(.65-9.71)
*p*	.25	.19
*General prerecorded message*		
Odds ratio	.43	.40
(95% CI)	(.12-1.57)	(.07-2.21)
*p*	.20	.29
*Staff guidance*		
Odds ratio	.33*	.38
(95% CI)	(.12-.90)	(.09-1.53)
*p*	.03	.17
*Live announcement*		
Odds ratio	1.01	1.24
(95% CI)	(.24-4.24)	(.18-8.39)
*p*	.99	.83
*Perceivable threat*		
Odds ratio	.39	.39
(95% CI)	(.15-1.02)	(.11-1.40)
*p*	.06	.15
Cox & Snell *R^2^*	.117	.129
Nagelkerke *R^2^*	.16	.17

*Note.* *** *p* < .001; ** *p* < .01; * *p* < .05. The odds ratios are presented with their 95% confidence intervals, meaning there is a .95 probability that the odds ratio will lie within this interval.

## Appendix

Here we present the results of the delayed response (quantitative) variable. Based on previous research and different ways to measure response times, we analyzed the response times quantitatively in combination with a qualitative layer as mentioned in the main text (Gwynne and Boyce, 2016, Purser and Bensilum, 2001). For completeness, we also include the results for the purely quantitative variable in this appendix.

## Results: Delayed response (quantitative)

The distribution of response times in the emergency videos reviewed are shown in Fig. 1, for 78 of the 126 videos it was possible to classify (Table 4). The distributions for the majority of observed people are shown. Statistical analyses were performed for the dichotomized response time, where a response time of 30 s or more indicates a delayed response. The Chi-square tests show that this risk behavior is more likely when the evacuation alarm is present, but not more or less likely when staff guidance, a general prerecorded message, or a live announcement were present (Table 5). Taking the context into account, this risk behavior was less likely when a perceivable threat was present (Table 5). The logistic regression shows that this risk behavior was not more or less likely when the communication strategies were present or when taking the context into account (Table 6).

## Footnotes

1 Although we flagged videos in which filming occurred while stopping or moving against the recommended evacuation direction, we noted that filming typically did not appear to be a hindrance to others in the videos. We found no instances in which the person filming was blocking an exit. Even when many people were filming a performance on a stage that then caught fire (see video of the opening of the King Kong movie), it did not seem to prevent others from evacuating.
